# Manifestations of COVID-19 in pregnant women with focus on gastrointestinal symptoms: a systematic review 

**Published:** 2020

**Authors:** Somayeh Makvandi, Sara Ashtari, Amir Vahedian-Azimi

**Affiliations:** 1 *Department of Midwifery, School of Nursing and Midwifery, Islamic Azad University AhvazBranch, Ahvaz, Iran*; 2 *Gastroenterology and Liver Diseases Research Center, Research Institute for Gastroenterology and Liver Diseases, Shahid Beheshti University of Medical Sciences, Tehran, Iran*; 3 *Trauma Research Center, Nursing Faculty, Baqiyatallah University of Medical Sciences, Tehran, Iran *

**Keywords:** COVID-19, Novel Coronavirus Infection, Pregnancy, Diarrhea, Gastrointestinal Tract

## Abstract

**Aim:**

This review study was conducted to evaluate the symptoms of COVID-19 in pregnant women with a focus on gastrointestinal symptoms.

**Background:**

COVID-19 is a fatal respiratory disease caused by a novel coronavirus that quickly became a pandemic. Although the main symptoms of this disease include respiratory symptoms, gastrointestinal manifestations have also been observed in some patients suffering from COVID-19. Pregnant women are among the most vulnerable groups in the community to infectious diseases.

**Methods:**

Scientific databases were searched for articles published up to May 8, 2020. Any type of study investigating the manifestations of COVID-19 in pregnant women was included. Symptoms of the disease in pregnant women with an emphasis on gastrointestinal symptoms were assessed.

**Results:**

The search resulted in 852 titles and abstracts, which were narrowed down to 43 studies involving 374 women. The most common symptoms of patients were fever (59.1%) and cough (48.4%), respectively. Gastrointestinal symptoms included diarrhea (4.5%), abdominal pain (1.6%), nausea (0.8%), and loss of appetite (0.3%), respectively. In studies on pregnant women with gastrointestinal symptoms, 13 fetal abortions occurred, most of which were induced abortions due to the risks posed by COVID-19.In thirty cases, and infected pregnant women reported a history of chronic pregnancy-related diseases.

**Conclusion:**

COVID-19 in pregnant women, similar to the general population, can present with gastrointestinal manifestations. The gastrointestinal tract can be a potential route for infection with the novel coronavirus.

## Introduction

 Coronaviruses comprise a large family of viruses that, according to evidence, can cause diseases such as the common cold, to more severe diseases such as the Middle East respiratory syndrome (MERS), or even more severe, such as severe acute respiratory syndrome (SARS) (1). In December 2019, a new type of coronavirus that had not previously been seen in humans was identified in Wuhan, China. The novel virus, called SARS-CoV-2, has been linked to a new respiratory syndrome called COVID-19 (2). This disease spread rapidly, resulting in epidemics in China and reports of multiple cases worldwide, and has been declared by the World Health Organization as a pandemic situation and the sixth public health emergency of international concern (3). 

The knowledge and understanding of COVID-19 are advancing rapidly, but evidence specifically focused on pregnant women is limited. They appear to be one of the most vulnerable groups to COVID-19. Physiological changes during pregnancy in the pulmonary system, such as increased oxygen consumption, increased minute ventilation, and decreased lung capacity, increase the risk of developing severe respiratory diseases in pregnant women. In addition, suppression of immune responses during pregnancy increases the risk of infections (4, 5).

Although the main symptoms of this disease include respiratory symptoms such as fever, dry cough, fatigue, and shortness of breath(3, 6), according to the evidence, gastrointestinal manifestations have also been observed in some patients suffering from COVID-19. One hypothesis in this regard is the use by the coronavirus of human angiotensin-converting enzyme 2 (ACE-2) receptors located on intestinal cells, hepatocytes, and cholangiocytes (7). Sellevoll et al. presented a woman with suspected cholecystitis who was admitted with acute abdominal pain and had no symptoms of respiratory infection, but eventually, a coronavirus test was positive (8). Findings from some studies on pregnant women infected by SARS-CoV-2 have indicated gastrointestinal symptoms such as abdominal pain, nausea, vomiting, and diarrhea in addition to respiratory symptoms (9, 10).

This review study was conducted to evaluate the symptoms of COVID-19 in pregnant women with a focus on gastrointestinal symptoms. 

**Figure1:**
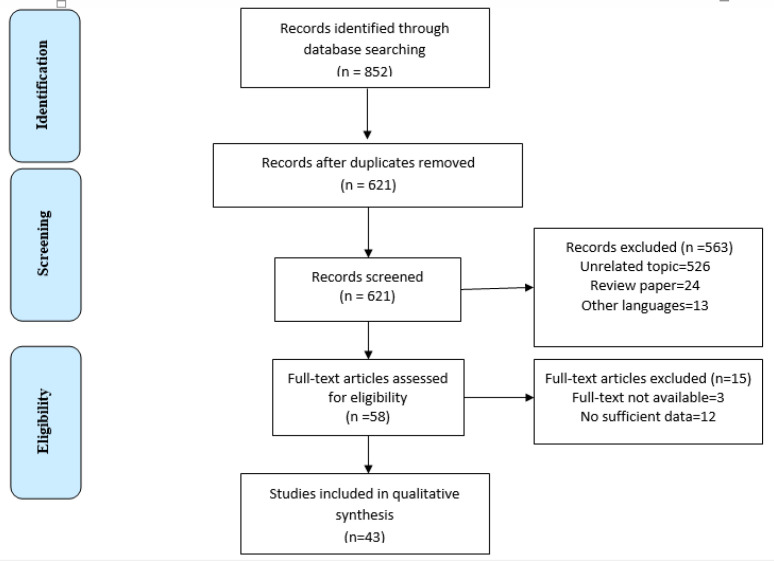
PRISMA Flow Diagram of the study

## Methods

Two investigators searched the Cochrane Library, Cochrane Central Register of Controlled Trials (CENTRAL), MEDLINE/PubMed, and SCOPUSfor articles published up to May 8, 2020. The search strategy was: (coronavirus OR “coronavirus disease-19” OR “COVID-19” OR “2019-nCoV disease” OR “2019 novel coronavirus infection” OR “2019-nCoV infection” OR “COVID-19 pandemic” OR “2019 novel coronavirus disease”) AND (pregnant* OR Childbearing OR Prenatal).

Any type of study with original data involving pregnant women infected bySARS-CoV-2in any trimester of pregnancy was included in the study. Articles in languages other than English, those that did not have enough data to answer the research question, and those that were only abstract were excluded. Our outcomes were any type of symptoms of COVID-19, mode of delivery, history of chronic basic diseases, vertical transmission, and maternal complications.

In the first step, two researchers independently examined the search output on each database. They reviewed the titles and abstracts of the articles and removed irrelevant studies. In the second step, the full text of the remaining articles was carefully reviewed. Any disagreement was resolved through discussion. The data retrieval tool was a researcher-made form that included data such as the name of the first author; country; sample size; pregnancy characteristics; general, respiratory, and gastrointestinal symptoms at the time of hospitalization or in the days following; mode of delivery; the history of chronic basic diseases; rate of vertical transmission; and maternal complications. 

Throughout the preparation of this article, the researchers adhered to the ethical principles in research; they never manipulated data to achieve their advantages and avoided plagiarism. 

## Results

Figure 1 shows the PRISMA flowchart for study inclusion and exclusion. A total of 852 studies corresponding to our search strategy were identified. Throughout data reduction, 563 irrelevant records were excluded based on the title and abstract review. The full-text articles for the remaining 58 articles were retrieved. After review of the full-text articles, 15 articles were excluded and 43met our inclusion criteria for the review. The characteristics of these studies are presented in Table 1. 

**Participants and settings**

A total of 374 samples were involved, ranging from 1 to 118 per study. In general, the gestational age in most women was 37 weeks or more (n=161, 62.9%). Twenty-three studies were undertaken in China(9, 11-32), seven studies were conducted in the USA(33-39), four studies in Italy(40-43), three in Iran(10, 44, 45), and one study each in Peru(46), Portugal(47), Turkey(48), Sweden(49), Central America(50), and Korea(51).

**Manifestations of COVID-19**

The symptoms of COVID-19 were classified into three categories: general, respiratory, and gastrointestinal. The most common general symptoms of COVID-19 in the studied pregnant women were fatigue (n=36, 9.6%) and myalgia (n=23, 6.1%), respectively. The most common respiratory symptoms in pregnant women included fever (n=221, 59.1%), cough (n=181, 48.4%), and dyspnea or shortness of breath (n=47, 12.5%), respectively. Among the gastrointestinal symptoms associated with novel coronavirus infection, diarrhea (n=17, 4.5%) and abdominal pain (n=6, 1.6%) were more common than others.

Focus on studies of pregnant women with gastrointestinal symptoms of COVID-19:

In 12 studies (n=259), pregnant women reported gastrointestinal symptoms caused by COVID-19 (Table 2). Most of these studies were conducted in China (66.6%). Gastrointestinal symptoms included diarrhea, abdominal pain, nausea, and loss of appetite. In studies of pregnant women with gastrointestinal symptoms, 159 cesarean sections (61.4%) and 40 vaginal deliveries (15.4%) were performed. There were also 13 abortions, most of which were induced abortions due to the risks posed by COVID-19.

In thirty cases, infected pregnant women reported a history of chronic or pregnancy-related diseases. In order of frequency, they were gestational diabetes mellitus (26.6%), anemia (16.6%), diabetes mellitus (13.3%), hypothyroidism (13.3%), obesity (10%), high blood pressure (6.6%), polycystic ovarian disease (6.6%), and hepatitis B (6.6%). The vertical transmission rate was 7.6%. No maternal deaths were reported. The need for mechanical ventilation was 0.9%.

## Discussion

To the best of our knowledge, this is the first study that focused on the gastrointestinal symptoms of COVID-19 in pregnant women. We did not find any similar study on pregnant women focusing on gastrointestinal symptoms of COVID-19 to compare with our findings. In the present study, the most common gastrointestinal symptoms of COVID-19 in pregnant women were diarrhea (4.5%) and abdominal pain (1.6%), respectively. Evidence suggests that approximately 2% to 33% of patients suffered from diarrhea as one of the symptoms of COVID-19 (53, 54).Chan et al. stated that 10.6% of patients with SARS and 30% of patients with MERS complained of diarrhea(55).It has been shown that MERS coronavirus can survive in simulated gastrointestinal juice and can cause intestinal infections(55).Some studies have shown the presence 

**Table1:** Characteristics of Studies including COVID-19 in Pregnant Women

First Author's Name		n		Country	Gestational Age				Symptoms of COVID-19																				
									General Symptoms						Respiratory Symptoms										Gastrointestinal Symptoms				
					Preterm (<37 weeks)		Term (≥37 weeks)		Fatigue		**Myalgia**		Headache		**F** **ever**		Cough		**Dyspnea or shortness of breath.**		**Nasal congestion**		Sputum		Diarrhea		Abdominal pain	Nausea	Loss of appetite
Alzamora,MC(46)		1		Peru	1		-		1		1		-		**1**		-		1		-		-		-		-	-	-
Browne, PC(33)		1		USA	1		-		-		1		-		**1**		1		-		-		-		-		-	-	-
Buonsenso, D(40)		4		Italy	3		1		-		-		-		**3**		4		1		-		-		-		-	-	-
Cao, D(11)		10		China	3		7		1		-		-		**7**		1		-		-		-		-		-	-	-
Carosso, A(41)		1		Italy	-		1		-		-		-		**1**		1		-		-		-		-		-	-	-
Chen, L(9)		118		China	NM				-		19		-		-		**84**				7				-			-	-
Chen, R(12)		17		China	3		14		1		-		-		**4**		4		1		-		-		1		-	-	-
Chen, S(13)		5		China	-		5		-		-		-		**5**		2		-		-		1		-		-	-	-
Chen, Y(14)		4		China	-		4		2		-		2		**3**		2		-		-		-		-		-	-	-
De Castro, A(34)		1		USA	1		-		1		-		1		1		-		-		-		-		-		-	-	-
Fan, C(15)		2		China	1		1		-		-		-		2		-		-		2		-		-		-	-	-
Ferrazzia, E(42)		42		Italy	12		30				7				20		18		8		-		-		2		-	-	-
Gidlöf, S(49)		1		Sweden	1		-		-		1		1		1		-		-		-		-		-		-	-	-
Hantoushzadeh,S(44)		9		Iran	6		3		-		4		-		9		9		6		-		-		-		-	-	-
Hirshberg, A(35)		5		USA	5		-		1		-		1		5		3		5		-		-		-		-	-	-
Iqbal, S(36)		1		USA	-		1		-		1		-		1		1		-		-		-		-		-	-	-
Juusela, A(37)		2		USA	1		1		-		-		-		1		-		1		-		-		-		-	1	-
Kalafat, E(48)		1		Turkey	1		-		-		-		-		-		1		1		-		-		-		-		-
Karami, P(45)		1		Iran	1		-		-		1		-		1		1		-		-		-		-		-	-	-
Kelly, JC(38)		1		USA	1		-		-		-		-		1		1		1		-		-		-		-	1	-
Khan, S-1(16)		3		China	1		2		-		-		-		2		3		-		-		-		-		-	-	-
Khan, S-2(17)		17		China	3		14		-		-		-		3		6		2		2		1		3		-	-	-
Lee, DH(51)		1		Korea	-		1		-		-		-		1		1		-		-		1		-		-	-	-
Li, J(18)		1		China	1		-		-		-		-		1		1		1		-		-		-		-	-	-
Li, Y(19)		1		China	1		-		-		-		-		-		1		-		-		-		-		-	-	-
Liao, J(20)	10		China		1	9		-		-		-		5		3		-		-		-		-		-		-	-
Liu,D(21)	15		China		7	8		4		3		-		13		9		1		-		-		1		-		-	-
Liu, Y(22)	13		China		10	3		4		-		-		10		2		3		-		-		-		-		-	-
Lu, D(23)	1		China		-	1		-		-		-		-		-		-		-		-		-		-		-	-
Lyra, J(47)	1		Portugal		-	1		-		-		-		-		1		-		-		-		-		-		-	-
Martinelli, I(43)	1		Italy		1	-		-		-		-		-		-		1		-		-		-		-		-	-
Peng, Z(24)	1		China		1	-		1		-		-		1		-		1		-		-		-		-		-	-
Qiancheng, X(25)	28		China		9	19		-		1		-		5		7		2		-		-		-		5		-	-
Schnettler, WT(39)	1		USA		1	-		-		1		-		1		1		1		-		-		-		-		-	-
Wang, S(26)	1		China		-	1		-		-		-		1		-		-		-		-		-		1		-	-
Wang,X(27)	1		China		1	-		-		-		-		1		-		-		-		-		-		-		-	-
Wu, C(28)	8		China		2	6		-		-		-		4		-		-		-		-		-		-		-	-
Wu, X(29)	23		China		6	17		-		-		-		4		6		-		1		-		-		-		-	-
Xiong, X(30)	1		China		1	-		-		-		-		1		1		-		-		-		-		-		-	-
Yu, N(31)	7		China		-	7		-		-		-		6		1		1		-		-		1		-		-	-
Zamaniyan, M(10)	1		Iran		1	-		-		1		-		1		1		1		-		-		-		-		1	1
Zambrano, LI(50)	1		Central America		1	-		1		1		-		1		1		-		-		-		-		-		-	-
Zhu, H(32)	10		China		6	4		-		-		-		9		5		-		-		-		1		-		-	-
Total	374		-		95	161		36		23		12		221		181		47		5		3		17		6		3	1

**NM: Not Mentioned**

of SARS-CoV-2 in the stool of patients suffering from COVID-19 (54, 56). Such evidence suggests that the gastrointestinal tract may be one of the potential routes of SARS-CoV-2 invasion and transmission, and there is also the possibility of oral-fecal transmission, indicating the need for effective revision of diagnostic and diagnostic guidelines.

According to our results, the second most common gastrointestinal symptom in infected pregnant women was abdominal pain (1.6%). Studies on non-pregnant populations have shown that abdominal pain as one of the symptoms of COVID-19 was rated from 2.2% to 5.8% (55, 57). In some studies, liver damage caused by SARS-CoV-2, impaired liver function tests, and increased bilirubin levels have also been reported in infected patients (58). Therefore, the presence of gastrointestinal symptoms with or without respiratory symptoms may be a warning sign of COVID-19.

**Table2:** Characteristics of Studies with Gastrointestinal Symptoms of COVID-19 in Pregnant Women

First Author's Name	N	Country	GA (weeks)		Gastrointestinal Symptoms of COVID-19				Method of Birth					History of Chronic Basic Diseases									Vertical transmission	Maternal Complications	
			Preterm	Term	Diarrhea	Abdominal pain	Nausea	Loss of appetite	Vaginal delivery	Cesarean section	Pregnant at End of Study		Abortion	DM	GDM	High blood pressure	Obesity	PCOD	Hypothyroidism	Anemia	Hepatitis B	Not Mentioned		Death	Mechanical ventilation
Chen, L (9)	118	China	NM		8		-	-	5	63	41	9		-	-	-	-	-	-	-	-	+	0/8	-	-
Chen, R (12)	17	China	3	14	1	-	-	-	-	17	-	-		2	-	1	-	-	-	5	-	-	-	-	-
Ferrazzia, E (42)	42	Italy	12	30	2	-	-	-	24	18	-	-		-	6	-	-	-	-	-	-	-	3/42	-	NM
Juusela, A (37)	2	USA	1	1	-	-	1	-	2	-	-	-		-	1	-	2	1	-	-	-	-	NM	-	1
Kelly, JC (38)	1	USA	1	-	-	-	1	-	-	1	-	-		-	-	-	1	-	-	-	-	-	-	-	1
Khan, S-2 (17)	17	China	3	14	3	-	-	-	-	17	-	-		-	-	-	-	-	-	-	-	+	2/17	-	-
Liu,D (21)	15	China	7	8	1	-	-	-	1	10	4	-		-	1	-	-	-	-	-	-	-	NM	-	-
Qiancheng, X (25)	28	China	9	19	-	5	-	-	5	17	2	4		2	-	1	-	-	1	-	2	-	0/23	-	-
Wang, S (26)	1	China	-	1	-	1	-	-	-	1	-	-		-	-	-	-	-	1	-	-	-	1	-	-
Yu, N (31)	7	China	-	7	1	-	-	-	-	7	-	-		-	-	-	-	1	1	-	-	-	1/3	-	-
Zamaniyan, M (10)	1	Iran	1	-	-	-	1	1	-	1	-	-		-	-	-	-	-	1	-	-	-	1	-	-
Zhu, H (32)	10	China	6	4	1	-		-	3	7	-	-		-	-	-	-	-	-	-	-	+	0/9	-	-
Total	259	-			17	6	3	1	40	159	47	13		4	8	2	3	2	4	5	2	-	8/104	-	2

DM: Diabetes Mellitus; GMM: Gestational Diabetes Mellitus; PCOD: Polycystic Ovarian Disease; NM: Not Mentioned

Although the main mechanism that explains the presence of gastrointestinal manifestations caused by COVID-19is unknown; one possible mechanism is the ability of SARS-CoV-2, like SARS-CoV, to bind to ACE-2 receptors, which are abundantly expressed in the gastrointestinal tract (57). Pan et al. stated that SARS-CoV-2 itself may cause disorders of the intestinal flora, which could result in gastrointestinal manifestations (59). Another possible mechanism is that changes in the composition and function of the gastrointestinal tract and respiratory tract flora can affect each other. This effect is called the “gut-lung axis" and can help explain why patients with COVID-19 often have gastrointestinal symptoms (60).

A significant number of pregnant women in whom gastrointestinal symptoms were among the manifestations of COVID-19 were predisposed to underlying chronic diseases. According to increasing evidence, the risk of developing COVID-19 and worsening of the condition of those with a chronic disease such as high blood pressure, lung disease, kidney problems, diabetes, or heart disease is higher than it is in healthy people(61).Therefore, it is recommended that pregnant women suffering from underlying diseases quarantine at home, because the weakening of their immune system and specific physiological changes related to pregnancy in interaction with the underlying disease increase the risk of infection by SARS-CoV-2and its serious complications.

The current study found that COVID-19 gastrointestinal symptoms were lower in pregnant women than in the non-pregnant populations reported in some studies (59). It seems that because the disease first appeared with respiratory symptoms, these have been the focus of most studies, and gastrointestinal symptoms have been neglected in some lower quality studies. Non-English language studies were also excluded from the current study, which may be a factor in this finding. To achieve more reliable and accurate results, more original, higher quality studies should be conducted.

Covid-19 in pregnant women, similar to the general population, can present with gastrointestinal manifestations. The gastrointestinal tract can be a potential route for infection with the novel coronavirus. According to the evidence, there is also the possibility of oral-fecal transmission. In pregnant women, as in the non-pregnant population, gastrointestinal symptoms in addition to the respiratory symptoms of COVID-19 should be considered.
